# Inappropriate prescribing and adverse drug events in older people

**DOI:** 10.1186/1471-2318-9-5

**Published:** 2009-01-28

**Authors:** Hilary J Hamilton, Paul F Gallagher, Denis O'Mahony

**Affiliations:** 1 Department of Geriatric Medicine, Cork University Hospital, Wilton, Cork, Ireland

## Abstract

Inappropriate prescribing (IP) in older patients is highly prevalent and is associated with an increased risk of adverse drug events (ADEs), morbidity, mortality and healthcare utilisation. Consequently, IP is a major safety concern and with changing population demographics, it is likely to become even more prevalent in the future. IP can be detected using explicit or implicit prescribing indicators. Theoretically, the routine clinical application of these IP criteria could represent an inexpensive and time efficient method to optimise prescribing practice. However, IP criteria must be sensitive, specific, have good inter-rater reliability and incorporate those medications most commonly associated with ADEs in older people. To be clinically relevant, use of prescribing appropriateness tools must translate into positive patient outcomes, such as reduced rates of ADEs. To accurately measure these outcomes, a reliable method of assessing the relationship between the administration of a drug and an adverse clinical event is required. The Naranjo criteria are the most widely used tool for assessing ADE causality, however, they are often difficult to interpret in the context of older patients. ADE causality criteria that allow for the multiple co-morbidities and prescribed medications in older people are required. Ultimately, the current high prevalence of IP and ADEs is unacceptable. IP screening criteria need to be tested as an intervention to assess their impact on the incidence of ADEs in vulnerable older patients. There is a role for IP screening tools in everyday clinical practice. These should enhance, not replace good clinical judgement, which in turn should be based on sound pharmacogeriatric training.

## Background

Older patients often have numerous co-morbidities for which they are prescribed multiple medications, thereby increasing the risk of adverse drug events (ADEs) [1]. This risk is compounded by age-related changes in physiology and body composition, which influence drug handling and response [2]. Furthermore, there is marked heterogeneity in health status and functional capacity in older people, often making prescribing decisions complex and challenging [2-4]. Evidence suggests that suboptimal or inappropriate prescribing (IP) is highly prevalent in older people and is associated with an increased risk of ADEs, increased morbidity, mortality and healthcare utilisation [5-9]. With changing worldwide population demographics and an aging population, IP in older people is becoming a global healthcare concern [5].

IP encompasses the use of medicines that pose more risk than benefit, particularly where safer alternatives exist. IP also includes the misuse of medicines (inappropriate dose or duration), the prescription of medicines with clinically significant drug-drug and drug-disease interactions, and importantly, the under-use of potentially beneficial medications [5]. IP can be detected using explicit (criterion-based) or implicit (judgement-based) prescribing indicators. Beers' criteria are the most widely cited explicit tool and have dominated the international literature since their development in the U.S. in 1991 [10]. They consist of two lists of medications to be avoided in older people, (a) independent of diagnosis, and (b) considering diagnosis, and do not address under-prescribing, drug-drug interactions or drug class duplication. They were originally designed for older nursing home residents, but were revised in 1997 [11] and 2002 [12] to be universally applicable to older patients. More recently, the STOPP (Screening Tool of Older Persons' potentially inappropriate Prescriptions) criteria were validated in a European setting [13]. STOPP criteria (see additional file 1) are arranged according to physiological systems for ease of use and include reference to drug class duplication, drug-drug and drug-disease interactions. They are uniquely designed for use alongside the START (Screening Tool to Alert doctors to the Right Treatment) criteria, which highlight under-prescription or omission of clinically indicated, evidence-based medications [14], thereby addressing more domains of prescribing appropriateness than Beers' criteria alone. Explicit criteria have been criticised for having limited transferability between countries due to variations in regional prescribing patterns and drug availability [5]. Explicit criteria must also be regularly updated in line with evolving clinical evidence.

The Medication Appropriateness Index (MAI) [15] is an implicit tool which measures prescribing appropriateness according to ten criteria including indication, effectiveness, dose, administration, drug-drug and drug-disease interactions and cost. It does not address under-prescribing. Clinical expertise is required to apply some of the criteria, resulting in variable inter-rater reliability. Consequently, the MAI is predominantly used as a research tool.

## Prevalence of inappropriate prescribing in the elderly

IP is highly prevalent in older people, with up to 24% of community-dwelling patients [16] and 40% of nursing home residents in the United States [17] regularly receiving at least one potentially inappropriate medicine (PIM) according to Beers' criteria. IP prevalence is somewhat lower in Europe, though comparison between studies is limited by differing methodologies. Under-prescribing is even more widespread – a recent study found that 58% of older patients do not receive one or more clinically indicated medications according to START criteria [14]. Risk factors for IP include older age, polypharmacy and multiple attending physicians and pharmacists [5]. IP is associated with increased morbidity, mortality and healthcare cost, largely because of an increased prevalence of ADEs [5].

## Adverse drug events and inappropriate prescribing

ADEs are defined as any injury resulting from drug therapy – from appropriate care, or unsuitable or suboptimal care [18]. ADEs include adverse reactions during normal use of a medicine, and any harm due to medication error whether of omission or commission. Up to 35% of community-dwelling older people experience ADEs each year [19], the incidence being even higher amongst nursing home residents [8]. Up to 30% of hospital admissions in older people are related to ADEs [20]. The clinical relevance of IP relates to its association with negative outcomes including preventable ADEs. Therefore, regular application of IP screening criteria should, hypothetically, reduce the prevalence of ADEs and related morbidity. To accurately measure such outcomes, reliable assessment of the relationship between drug administration and adverse clinical event is required, both in terms of causality and preventability. The Naranjo criteria are often used to assess ADE causality (see additional file 2), with inter-rater agreement scores superior to subjective clinical judgement [21]. However, they can be difficult to interpret in the context of older patients with multiple co-morbidities and medications. ADEs in older patients often present with non-specific symptoms or geriatric syndromes such as cognitive impairment or falls e.g. a fall may be related to osteoarthritis or poor visual acuity as well as prescription of a medication that increases falls risk such as a benzodiazepine. The causal association can also be weakened as the Naranjo criteria evaluate drugs individually and do not address drug-drug interactions (see additional file 2).

The Hallas criteria classify ADEs as preventable, probably preventable, probably not preventable or definitely not preventable [22]. Preventable ADEs include those arising from the prescription of PIMs and suboptimal monitoring and dose adjustment. Non-preventable ADEs include allergic or idiosyncratic reactions.

The ultimate aim of IP screening tools is to optimise prescribing appropriateness and reduce negative outcomes including preventable ADEs. Therefore, the medications listed by explicit IP tools should be those most commonly associated with preventable ADEs in older people. Some studies have demonstrated no increased risk of ADEs in patients receiving Beers' criteria medications [23-25]. Some also conclude that Beers' criteria PIMs account for only a small proportion of ADEs in older patients [25,26]. However, interpretation of such studies is difficult as many were retrospective and lacked clinical detail, thereby resulting in incomplete application of Beers' criteria. Furthermore, many did not use rigorous ADE causality and preventability criteria. It is possible that Beers' criteria simply do not list those medications most commonly associated with preventable ADEs in older people, as suggested by a recent Irish study which reported that 12% of hospital admissions were related to ADEs resulting from STOPP criteria PIMs, with only 6% resulting from Beers' criteria PIMs [27].

Other interventions that optimise prescribing appropriateness include comprehensive geriatric assessment [28], clinical pharmacist intervention [29], prescriber education [30] and computerised decision support tools [31]. However, such interventions are resource intensive and not universally available. Consequently, there is a need for a simple, inexpensive and time-efficient screening tool which can be used routinely to guide prescribing practice and reduce the rate of IP in older patients. Such a tool should be sensitive, specific, include commonly encountered ADEs and have good inter-rater reliability. To be clinically relevant, use of such a screening tool must translate into positive clinical outcomes. Specific ADE causality assessment criteria for older people are also needed to measure the result of such interventions.

## Conclusion

Ultimately, the high prevalence of IP and preventable ADEs in older people is unacceptable, and represents a public health hazard likely to grow in tandem with ageing populations. Improved undergraduate and postgraduate training in geriatric pharmacotherapy is crucial. Though valid IP screening tools are desirable, they should enhance, not replace, clinical judgement. These screening tools need to be tested as an intervention in order to assess their impact on the incidence of ADEs in this vulnerable population.

## Pre-publication history

The pre-publication history for this paper can be accessed here:



## Supplementary Material

Additional file 1 **Screening tool of older people's potentially inappropriate prescriptions.** The following drug prescriptions are potentially inappropriate in persons aged ≥ 65 years of age. Click here for file

Additional file 2 **The Naranjo adverse drug reaction probability scale; To assess the adverse drug reaction, please answer the following questionnaire and give the pertinent score.** The Naranjo adverse drug reaction (ADR) probability scale. The Naranjo criteria classify the probability that an adverse event is related to drug therapy based on a list of weighted questions, which examine factors such as the temporal association of drug administration and event occurrence, alternative causes for the event, drug levels, dose – response relationships and previous patient experience with the medication. The ADR is assigned to a probability category from the total score as follows: *definite *if the overall score is 9 or greater, *probable *for a score of 5–8, *possible *for 1–4 and *doubtful *if the score is 0. The Naranjo criteria do not take into account drug-drug interactions. Drugs are evaluated individually for causality, and points deducted if another factor may have resulted in the adverse event, thereby weakening the causal association. Click here for file
