# Supplementary material for: Inappropriate prescribing and adverse drug events in older people
Source: BMC Geriatr. 2009 Jan 28;9:5. doi: 10.1186/1471-2318-9-5 (PMC2642820; doi:10.1186/1471-2318-9-5)
Supplement: Additional file 1 — Screening tool of older people's potentially inappropriate prescriptions. The following drug prescriptions are potentially inappropriate in persons aged ≥ 65 years of age. [file 1471-2318-9-5-S1.doc]

| **A. Cardiovascular System**  1. Digoxin at a long-term dose > 125µg/day with impaired renal function^*^ *(increased risk of toxicity).*  2. Loop diuretic for dependent ankle oedema only i.e. no clinical signs of heart failure *(no evidence of efficacy, compression hosiery usually more appropriate).*  3. Loop diuretic as first-line monotherapy for hypertension *(safer, more effective alternatives available).*  4. Thiazide diuretic with a history of gout *(may exacerbate gout).*  5. Non-cardioselective beta-blocker with Chronic Obstructive Pulmonary Disease (COPD) *(risk of bronchospasm)*.  6. Beta-blocker in combination with verapamil *(risk of symptomatic heart block).*  7. Use of diltiazem or verapamil with NYHA Class III or IV heart failure *(may worsen heart failure).*  8. Calcium channel blockers with chronic constipation *(may exacerbate constipation).*  9. Use of aspirin and warfarin in combination without histamine H2 receptor antagonist (except cimetidine because of interaction with warfarin) or proton pump inhibitor *(high risk of gastrointestinal bleeding).*  10. Dipyridamole as monotherapy for cardiovascular secondary prevention *(no evidence for efficacy).*  11. Aspirin with a past history of peptic ulcer disease without histamine H2 receptor antagonist or Proton Pump Inhibitor *(risk of bleeding).*  12. Aspirin at dose > 150mg day *(increased bleeding risk, no evidence for increased efficacy).*  13. Aspirin with no history of coronary, cerebral or peripheral vascular symptoms or occlusive arterial event *(not indicated).*  14. Aspirin to treat dizziness not clearly attributable to cerebrovascular disease *(not indicated)*.  15. Warfarin for first, uncomplicated deep venous thrombosis for longer than 6 months duration *(no proven added benefit).*  16. Warfarin for first uncomplicated pulmonary embolus for longer than 12 months duration *(no proven benefit).*  17. Aspirin, clopidogrel, dipyridamole or warfarin with concurrent bleeding disorder *(high risk of bleeding).*  **B. Central Nervous System and Psychotropic Drugs.**  1. Tricyclic antidepressants (TCA’s) with dementia *(risk of worsening cognitive impairment).*  2. TCA’s with glaucoma *(likely to exacerbate glaucoma).*  3. TCA’s with cardiac conductive abnormalities *(pro-arrhythmic effects).*  4. TCA’s with constipation *(likely to worsen constipation).*  5. TCA’s with an opiate or calcium channel blocker *(risk of severe constipation).*  6. TCA’s with prostatism or prior history of urinary retention *(risk of urinary retention).*  7. Long-term (i.e. > 1 month), long-acting benzodiazepines e.g. chlordiazepoxide, fluazepam, nitrazepam, chlorazepate and benzodiazepines with long-acting metabolites e.g. diazepam *(risk of prolonged sedation, confusion, impaired balance, falls).*  8. Long-term (i.e. > 1 month) neuroleptics as long-term hypnotics *(risk of confusion, hypotension, extra-pyramidal side effects, falls).*  9. Long-term neuroleptics ( > 1 month) in those with parkinsonism *(likely to worsen extra-pyramidal symptoms)*  10. Phenothiazines in patients with epilepsy *(may lower seizure threshold).*  11. Anticholinergics to treat extra-pyramidal side-effects of neuroleptic medications *(risk of anticholinergic toxicity).*  12. Selective serotonin re-uptake inhibitors (SSRI’s) with a history of clinically significant hyponatraemia *(non-iatrogenic hyponatraemia <130mmol/l within the previous 2 months).*  13. Prolonged use (> 1 week) of first generation antihistamines i.e. diphenydramine, chlorpheniramine, cyclizine, promethazine *(risk of sedation and anti-cholinergic side effects).*  **C. Gastrointestinal System**  1. Diphenoxylate, loperamide or codeine phosphate for treatment of diarrhoea of unknown cause (risk of delayed diagnosis, may exacerbate constipation with overflow diarrhoea, may precipitate toxic megacolon in inflammatory bowel disease, may delay recovery in unrecognised gastroenteritis).  2. Diphenoxylate, loperamide or codeine phosphate for treatment of severe infective gastroenteritis i.e. bloody diarrhoea, high fever or severe systemic toxicity (risk of exacerbation or protraction of infection)  3. Prochlorperazine (Stemetil) or metoclopramide with Parkinsonism *(risk of exacerbating Parkinsonism).*  4. PPI for peptic ulcer disease at full therapeutic dosage for > 8 weeks *(earlier discontinuation or dose reduction for maintenance/prophylactic treatment of peptic ulcer disease, oesophagitis or GORD indicated).*  5. Anticholinergic antispasmodic drugs with chronic constipation *(risk of exacerbation of constipation).*  **D. Respiratory System.**  1. Theophylline as monotherapy for COPD. *(safer, more effective alternative; risk of adverse effects due to narrow therapeutic index)*  2. Systemic corticosteroids instead of inhaled corticosteroids for maintenance therapy in moderate-severe COPD *(unnecessary exposure to long-term side-effects of systemic steroids).*  3. Nebulised ipratropium with glaucoma *(may exacerbate glaucoma).*  **E. Musculoskeletal System**  1. Non-steroidal anti-inflammatory drug (NSAID) with history of peptic ulcer disease or gastrointestinal bleeding, unless with concurrent histamine H2 receptor antagonist, PPI or misoprostol *(risk of peptic ulcer relapse).*  2. NSAID with moderate-severe hypertension (moderate: 160/100mmHg – 179/109mmHg; severe: ≥180/110mmHg) *(risk of exacerbation of hypertension).*  3. NSAID with heart failure *(risk of exacerbation of heart failure).*  4. Long-term use of NSAID (>3 months) for relief of mild joint pain in osteoarthtitis *(simple analgesics preferable and usually as effective for pain relief)*  5. Warfarin and NSAID together *(risk of gastrointestinal bleeding).*  6. NSAID with chronic renal failure^*^ *(risk of deterioration in renal function).*  7. Long-term corticosteroids (>3 months) as monotherapy for rheumatoid arthrtitis or osteoarthritis *(risk of major systemic corticosteroid side-effects).*  8. Long-term NSAID or colchicine for chronic treatment of gout where there is no contraindication to allopurinol *(allopurinol first choice prophylactic drug in gout)*  **F. Urogenital System**  1. Bladder antimuscarinic drugs with dementia *(risk of increased confusion, agitation).*  2. Bladder antimuscarinic drugs with chronic glaucoma *(risk of acute exacerbation of glaucoma).*  3. Bladder antimuscarinic drugs with chronic constipation *(risk of exacerbation of constipation).*  4. Bladder antimuscarinic drugs with chronic prostatism *(risk of urinary retention).*  5. Alpha-blockers in males with frequent incontinence i.e. one or more episodes of incontinence daily *(risk of urinary frequency and worsening of incontinence).*  6. Alpha-blockers with long-term urinary catheter *in situ* i.e. more than 2 months *(drug not indicated).*  **G. Endocrine System**  1. Glibenclamide or chlorpropamide with type 2 diabetes mellitus *(risk of prolonged hypoglycaemia).*  2. Beta-blockers in those with diabetes mellitus and frequent hypoglycaemic episodes i.e. ≥ 1 episode per month *(risk of masking hypoglycaemic symptoms).*  3. Oestrogens with a history of breast cancer or venous thromboembolism *(increased risk of recurrence)*  4. Oestrogens without progestogen in patients with intact uterus *(risk of endometrial cancer)*  *.*  **H. Drugs that adversely affect those prone to falls (≥ 1 fall in past three months)**  1. Benzodiazepines *(sedative, may cause reduced sensorium, impair balance).*  2. Neuroleptic drugs *(may cause gait dyspraxia, Parkinsonism).*  3. First generation antihistamines *(sedative, may impair sensorium).*  4. Vasodilator drugs known to cause hypotension in those with persistent postural hypotension i.e. recurrent > 20mmHg drop in systolic blood pressure *(risk of syncope, falls).*  5. Long-term opiates in those with recurrent falls *(risk of drowsiness, postural hypotension, vertigo).*  **I. Analgesic Drugs**  1. Use of long-term powerful opiates e.g. morphine or fentanyl as first line therapy for mild-moderate pain *(WHO analgesic ladder not observed).*  2. Regular opiates for more than 2 weeks in those with chronic constipation without concurrent use of laxatives *(risk of severe constipation).*  3. Long-term opiates in those with dementia unless indicted for palliative care or management of moderate/severe chronic pain syndrome *(risk of exacerbation of cognitive impairment).* J. Duplicate Drug Classes Any duplicate drug class prescription e.g. two concurrent opiates, NSAID’s, SSRI’s, loop diuretics, ACE inhibitors *(optimisation of monotherapy within a single drug class should be observed prior to considering a new class of drug). Excluding duplicate prescribing of inhaled beta2 agonists (long and short acting) for asthma or COPD* |
| --- |
| * Estimated GFR <50ml/min. |
