# Supplementary material for: Inappropriate prescribing and adverse drug events in older people
Source: BMC Geriatr. 2009 Jan 28;9:5. doi: 10.1186/1471-2318-9-5 (PMC2642820; doi:10.1186/1471-2318-9-5)
Supplement: Additional file 2 — The Naranjo adverse drug reaction probability scale; To assess the adverse drug reaction, please answer the following questionnaire and give the pertinent score. The Naranjo adverse drug reaction (ADR) probability scale. The Naranjo criteria classify the probability that an adverse event is related to drug therapy based on a list of weighted questions, which examine factors such as the temporal association of drug administration and event occurrence, alternative causes for the event, drug levels, dose – response relationships and previous patient experience with the medication. The ADR is assigned to a probability category from the total score as follows: definite if the overall score is 9 or greater, probable for a score of 5–8, possible for 1–4 and doubtful if the score is 0. The Naranjo criteria do not take into account drug-drug interactions. Drugs are evaluated individually for causality, and points deducted if another factor may have resulted in the adverse event, thereby weakening the causal association. [file 1471-2318-9-5-S2.doc]

| **Table 2:** The Naranjo adverse drug reaction probability scale; To assess the adverse drug reaction, please answer the following questionnaire and give the pertinent score | Yes | No | Do not know | Score |
| --- | --- | --- | --- | --- |
| 1. Are there previous *conclusive* reports on this reaction?  2. Did the adverse event occur after the suspected drug was administered?  3. Did the adverse reaction improve when the drug was discontinued or a *specific* antagonist was administered?  4. Did the adverse reaction reappear when the drug was readministered?  5. Are there alternative causes (other than the drug) that could have on their own caused the reaction?  6. Did the reaction reappear when a placebo was given?  7. Was the blood detected in the blood (or other fluids) in concentrations known to be toxic?  8. Was the reaction more severe when the dose was increased or less severe when the dose was decreased?  9. Did the patient have a similar reaction to the same or similar drugs in *any* previous exposure?  10. Was the adverse event confirmed by any objective evidence? | +1  +2  +1  +2  -1  -1  +1  +1  +1  +1 | 0  -1  0  -1  +2  +1  0  0  0  0 | 0  0  0  0  0  0  0  0  0  0 |  |
|  |  |  | Total |  |

**Table 2:** The Naranjo adverse drug reaction (ADR) probability scale. The Naranjo criteria classify the probability that an adverse event is related to drug therapy based on a list of weighted questions, which examine factors such as the temporal association of drug administration and event occurrence, alternative causes for the event, drug levels, dose – response relationships and previous patient experience with the medication. The ADR is assigned to a probability category from the total score as follows: *definite* if the overall score is 9 or greater, *probable* for a score of 5-8, *possible* for 1-4 and *doubtful* if the score is 0. The Naranjo criteria do not take into account drug-drug interactions. Drugs are evaluated individually for causality, and points deducted if another factor may have resulted in the adverse event, thereby weakening the causal association.
